# Association of *Fusobacterium* species in pancreatic cancer tissues with molecular features and prognosis

**DOI:** 10.18632/oncotarget.3109

**Published:** 2015-03-13

**Authors:** Kei Mitsuhashi, Katsuhiko Nosho, Yasutaka Sukawa, Yasutaka Matsunaga, Miki Ito, Hiroyoshi Kurihara, Shinichi Kanno, Hisayoshi Igarashi, Takafumi Naito, Yasushi Adachi, Mami Tachibana, Tokuma Tanuma, Hiroyuki Maguchi, Toshiya Shinohara, Tadashi Hasegawa, Masafumi Imamura, Yasutoshi Kimura, Koichi Hirata, Reo Maruyama, Hiromu Suzuki, Kohzoh Imai, Hiroyuki Yamamoto, Yasuhisa Shinomura

**Affiliations:** ^1^ Department of Gastroenterology, Rheumatology and Clinical Immunology, Sapporo Medical University School of Medicine, Sapporo, Japan; ^2^ Department of Medical Oncology, Dana-Farber Cancer Institute and Harvard Medical School, Boston, MA, USA; ^3^ Department of Gastroenterology, Teine Keijinkai Hospital, Sapporo, Japan; ^4^ Department of Pathology, Teine Keijinkai Hospital, Sapporo, Japan; ^5^ Department of Surgical Pathology, Sapporo Medical University School of Medicine, Sapporo, Japan; ^6^ Department of Surgery, Surgical Oncology and Science, Sapporo Medical University School of Medicine, Sapporo, Japan; ^7^ Department of Molecular Biology, Sapporo Medical University School of Medicine, Sapporo, Japan; ^8^ The Institute of Medical Science, The University of Tokyo, Tokyo, Japan; ^9^ Department of Gastroenterology and Hepatology, St. Marianna University School of Medicine, Kawasaki, Japan

**Keywords:** Fusobacterium, microbiota, pancreas, miR-31, survival

## Abstract

Recently, bacterial infection causing periodontal disease has attracted considerable attention as a risk factor for pancreatic cancer. *Fusobacterium* species is an oral bacterial group of the human microbiome. Some evidence suggests that *Fusobacterium* species promote colorectal cancer development; however, no previous studies have reported the association between *Fusobacterium* species and pancreatic cancer. Therefore, we examined whether *Fusobacterium* species exist in pancreatic cancer tissue. Using a database of 283 patients with pancreatic ductal adenocarcinoma (PDAC), we tested cancer tissue specimens for *Fusobacterium* species. We also tested the specimens for *KRAS*, *NRAS*, *BRAF* and *PIK3CA* mutations and measured microRNA-21 and microRNA-31. In addition, we assessed epigenetic alterations, including CpG island methylator phenotype (CIMP). Our data showed an 8.8% detection rate of *Fusobacterium* species in pancreatic cancers; however, tumor *Fusobacterium* status was not associated with any clinical and molecular features. In contrast, in multivariate Cox regression analysis, compared with the *Fusobacterium* species-negative group, we observed significantly higher cancer-specific mortality rates in the positive group (*p* = 0.023). In conclusion, *Fusobacterium* species were detected in pancreatic cancer tissue. Tumor *Fusobacterium* species status is independently associated with a worse prognosis of pancreatic cancer, suggesting that *Fusobacterium* species may be a prognostic biomarker of pancreatic cancer.

## INTRODUCTION

Pancreatic cancer is a highly aggressive malignancy, with < 50% patients surviving past 6 months after the diagnosis. Because chemotherapeutic options only marginally prolong life, the current mortality of patients with pancreatic cancer is nearly identical to its incidence [1–13]. Therefore, future studies are expected to elucidate the pathogenesis of pancreatic cancer and explore new possibilities for diagnostic and therapeutic approaches to the disease.

Recently, bacterial infection causing periodontal disease has attracted considerable attention as a risk factor for pancreatic cancer [3, 6, 7, 10, 14]. Individuals with periodontal disease have increased levels of markers of systemic inflammation, and oral bacteria can spread to the bloodstream [3, 6, 7, 15], gastrointestinal tract [16–19], liver [20, 21], or pancreas [22] and can even reach the brain [23]. Thus, oral bacteria may reach the pancreas too through the circulation or transductal transmission from the biliary tract and can contribute to pancreatic carcinogenesis by acting jointly with other pancreatic cancer risk factors that modulate inflammation and the immune response, e.g., obesity, smoking, and the *ABO* genetic variant [4, 6, 7].

*Fusobacterium* species (a group of non-spore-forming, anaerobic gram-negative bacteria) is an oral bacteria group of the human microbiome. The members of the *Fusobacterium* species are highly heterogeneous, and some of them have been recognized as opportunistic pathogens implicated not only in periodontitis [6, 7, 24] but also in inflammatory bowel diseases (IBD) [17–19], pancreatic abscess [22, 25], and hepatic abscess [20, 21, 25]. With regard to the association of *Fusobacterium* species with the pathogenesis of gastrointestinal cancer, metagenomic analyses involving whole-genome sequencing, transcriptome sequencing and 16S ribosomal RNA gene DNA sequencing have demonstrated enrichment of *Fusobacterium* species in colorectal cancer tissues [26–30]. Moreover, increased levels of *Fusobacterium* species are related to patient survival [29] and to specific molecular subsets of colorectal cancers [28].

Thus, accumulating evidence indicates that *Fusobacterium* species may play a role in gastrointestinal cancer; however, no previous studies have reported an association of *Fusobacterium* species with pancreatic cancer. Therefore, we examined whether *Fusobacterium* species exist in pancreatic cancer tissue and play a role in disease progression. To identify the role in pancreatic cancer, we also analyzed the association of tumor *Fusobacterium* species status with molecular characteristics, including epigenetic alterations and microRNA expression levels, and patient prognosis, using a database of 283 patients.

## RESULTS

### Detection of *Fusobacterium* species in cancer tissue specimens of pancreatic cancer

**Figure 1:**
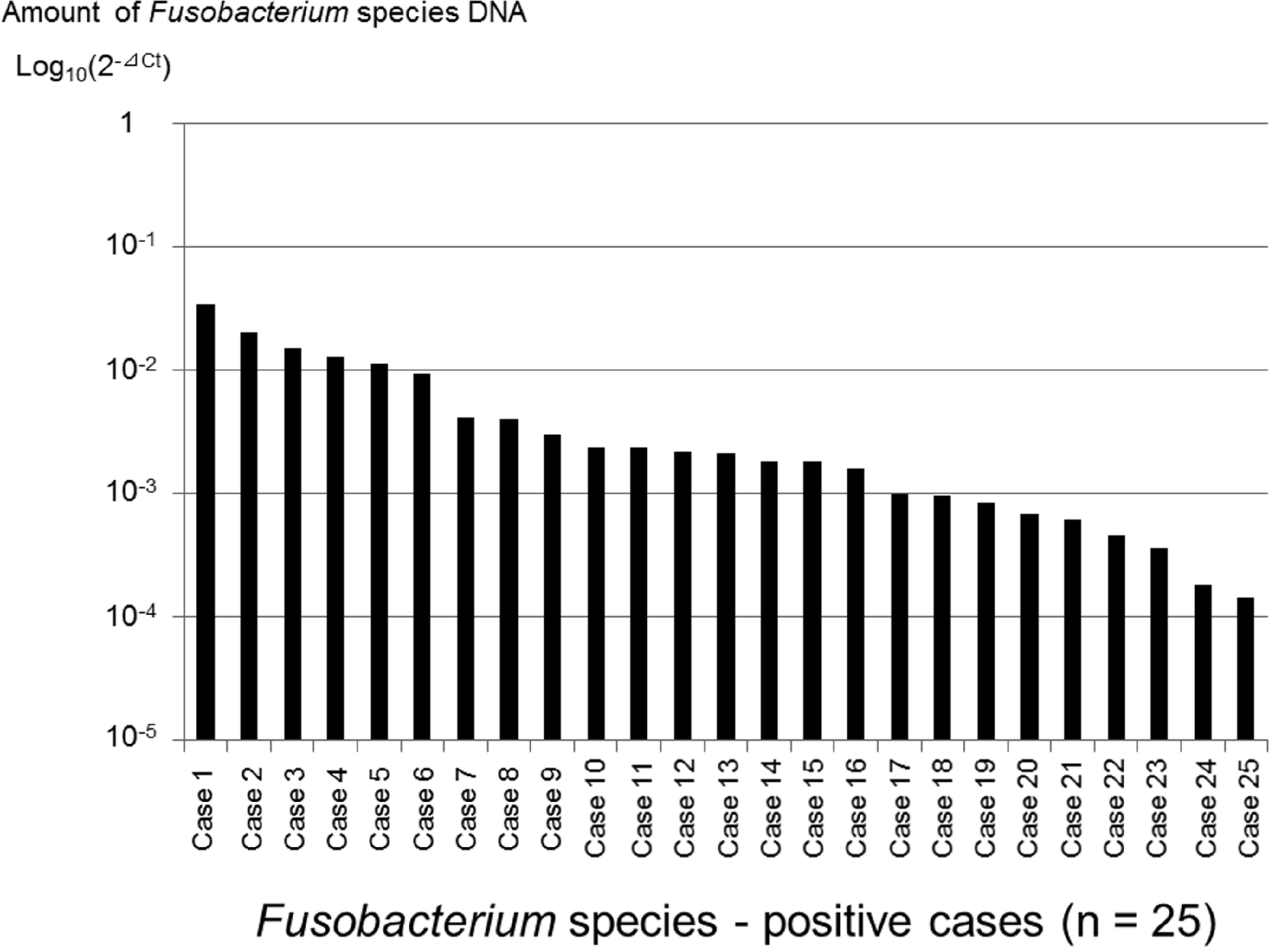
Distribution of *Fusobacterium* species-positive cases (*n* = 25) among pancreatic cancer tissue specimens The *Fusobacterium* species-positive cases were ranked according to the amount of Fusobacterium species DNA.

We assessed 302 formalin-fixed, paraffin-embedded (FFPE) tissue specimens of pancreatic cancers using the TaqMan Gene Expression Assay for Fusobacterium species and obtained 283 (94%) positive results. *Fusobacterium* species were detected in 8.8% (25/283) of the pancreatic cancer tissue specimens. Figure 1 shows DNA amounts (2^−ΔCt^) of the 25 *Fusobacterium* species-positive cases.

Using the tumor *Fusobacterium* species-positive group, we also tested paired specimens of normal pancreatic mucosal tissues for *Fusobacterium* species. Our data demonstrated that *Fusobacterium* species were detected in 28% (7/25) of the paired specimens of normal tissues.

### The association between tumor *Fusobacterium* species and clinical, pathological and molecular characteristics in pancreatic cancers

Table 1 summarizes clinical, pathological and molecular features of the 283 patients with pancreatic cancer, according to tumor *Fusobacterium* species status (positive versus negative). Tumor *Fusobacterium* species status was slightly but insignificantly associated with the female gender and high microRNA-21 (miR-21) expression levels. Neither microRNA-31 (miR-31) nor microRNA-143 (miR-143) correlate with tumor *Fusobacterium* species status.

**Table 1:** Clinical or molecular features of pancreatic cancers according to tumor *Fusobacterium* status

Clinical, pathological or molecular feature	Total	Tumor *Fusobacterium* species status		*P*
		Negative	Positive	
All cases	283	258	25	
Gender				
Male	161 (57%)	151 (59%)	10 (40%)	0.074
Female	122 (43%)	107 (42%)	15 (60%)	
Age (mean ± SD)	67.1 ± 9.1	67.3 ± 9.1	65.2 ± 8.4	0.25
Tumor location in pancreas				
Head	203 (72%)	187 (73%)	16 (64%)	0.12
Body	62 (22%)	57 (22%)	5 (20%)	
Tail	18 (6.4%)	14 (5.4%)	4(16%)	
Tumor size (cm)				
<2	44 (16%)	41 (16%)	3 (12%)	0.94
2–4	191 (68%)	174 (67%)	17 (68%)	
4–6	40 (14%)	36 (14%)	4 (16%)	
≥6	8 (2.8%)	7 (2.7%)	1 (4.0%)	
Lymph node invasion				
Negative	99 (35%)	89 (35%)	10 (40%)	0.58
Positive	184 (65%)	169 (66%)	15 (60%)	
Disease Stage(UICC classification)				
I	20 (7.1%)	18 (7.0%)	2 (8.0%)	0.10
II	248 (88%)	228 (88%)	20 (80%)	
III	10 (3.5%)	9 (3.5%)	1 (4.0%)	
IV	5 (1.8%)	3 (1.2%)	2 (8.0%)	
Year of diagnosis				
Prior to 2009	157 (55%)	139 (54%)	18 (72%)	0.076
2010–2013	126 (45%)	119 (46%)	7 (28%)	
*KRAS* gene				
Wild-type	88 (31%)	82 (32%)	6 (24%)	0.72
Codon 12/13 mutated	174 (61%)	157 (61%)	17 (68%)	
Codon 61/146 mutated	21 (7.4%)	19 (7.4%)	2 (8.0%)	
MicroRNA-21				
Low expression	213 (75%)	198 (77%)	15 (60%)	0.064
High expression	70 (25%)	60 (23%)	10 (40%)	
MicroRNA-31				
Low expression	213 (75%)	196 (76%)	17 (68%)	0.38
High expression	70 (25%)	62 (24%)	8 (32%)	
MicroRNA-143				
Low expression	70 (25%)	64 (25%)	6 (24%)	0.93
High expression	213 (75%)	194 (75%)	19 (76%)	
*MLH1* status				
Unmethylated	272 (96%)	248 (96%)	24 (96%)	0.98
Methylated	11 (3.9%)	10 (3.9%)	1 (4%)	
CIMP status				
CIMP-low/zero	249 (88%)	226 (88%)	23 (92%)	0.52
CIMP-high	34 (12%)	32 (12%)	2 (8.0%)	

Percentages (%) indicate the proportion of cases with a specific clinical, pathological, or molecular feature within a given dichotomous category of tumor *Fusobacterium* species status. The *p* values were calculated using t-test for age and χ^2^ test for gender; tumor location in pancreas; tumor size; lymph node invasion; year of diagnosis; *KRAS* mutation; expressions of microRNA-21, microRNA-31, and microRNA-143; *MLH1* methylation and CIMP status or Fisher's exact test for disease stage.

CIMP, CpG island methylator phenotype; SD, standard deviation; UICC, Unio Internationalis Contra Cancrum

### *Fusobacterium* species status in pancreatic cancer and patient survival

**Figure 2:**
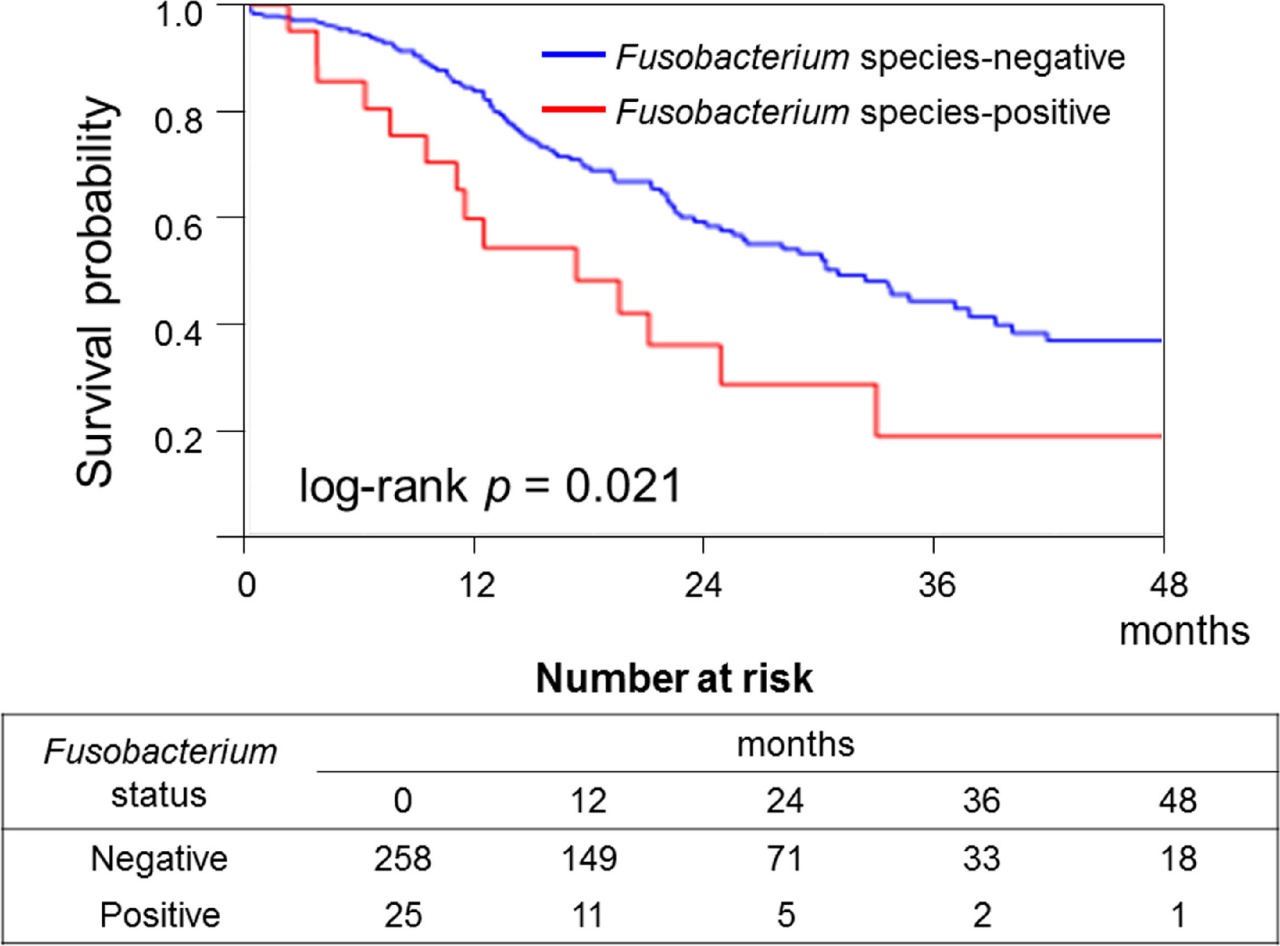
Kaplan–Meier curves of cancer-specific survival of patients with pancreatic cancer according to tumor *Fusobacterium* species status In the Kaplan–Meier analysis, significantly shorter survival was observed in the *Fusobacterium* species-positive group (log-rank *p* = 0.021).

During the follow-up of the 283 patients with pancreatic cancer, 109 patients died (all deaths were confirmed to be attributable to pancreatic cancer). The median follow-up period for cancer-specific survival was 30.3 months. In the Kaplan–Meier analysis, significantly shorter survival was observed in the *Fusobacterium* species-positive group [median cancer-specific survival (months): 17.2 versus 32.5; log-rank *p* = 0.021; Figure 2].

### The association of molecular alterations with patient survival in pancreatic cancer

*KRAS* mutation (codon 12, 13, 61, or 146) was detected in 69% of the 283 patients with pancreatic cancer (Table 1). *BRAF* (*V600E*), *NRAS* (codon 12, 13, or 61), and *PIK3CA* (exon 9 or 20) mutations were not found in these 283 cases. CpG island methylator phenotype (CIMP)-high status [three/four or more methylated promoters among *CACNA1G, CDKN2A*, *IGF2*, and *RUNX3*] was observed in 12% (34/283) of the pancreatic cancer cases (Table 1). Microsatellite instability (MSI)-high status was not detected among the 283 cases.

**Figure 3:**
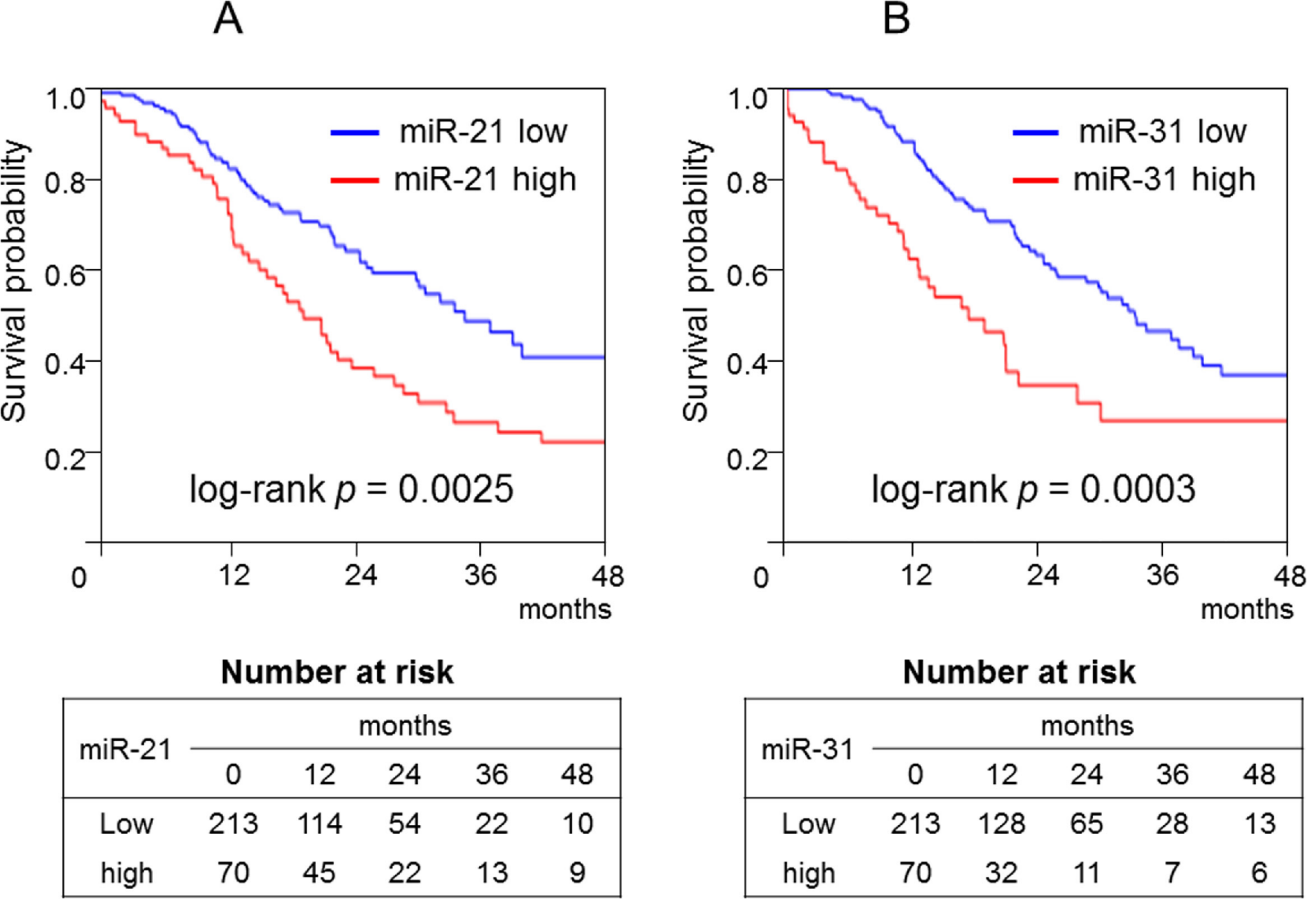
Kaplan–Meier curves of cancer-specific survival of patients with pancreatic cancer according to the amount of microRNA-21 (A) or microRNA-31 (B) In the Kaplan–Meier analysis, high microRNA-21 (log-rank *p* = 0.0025) and microRNA-31 expression levels (log-rank *p* = 0.0003) significantly correlated with shorter survival.

**Figure 4:**
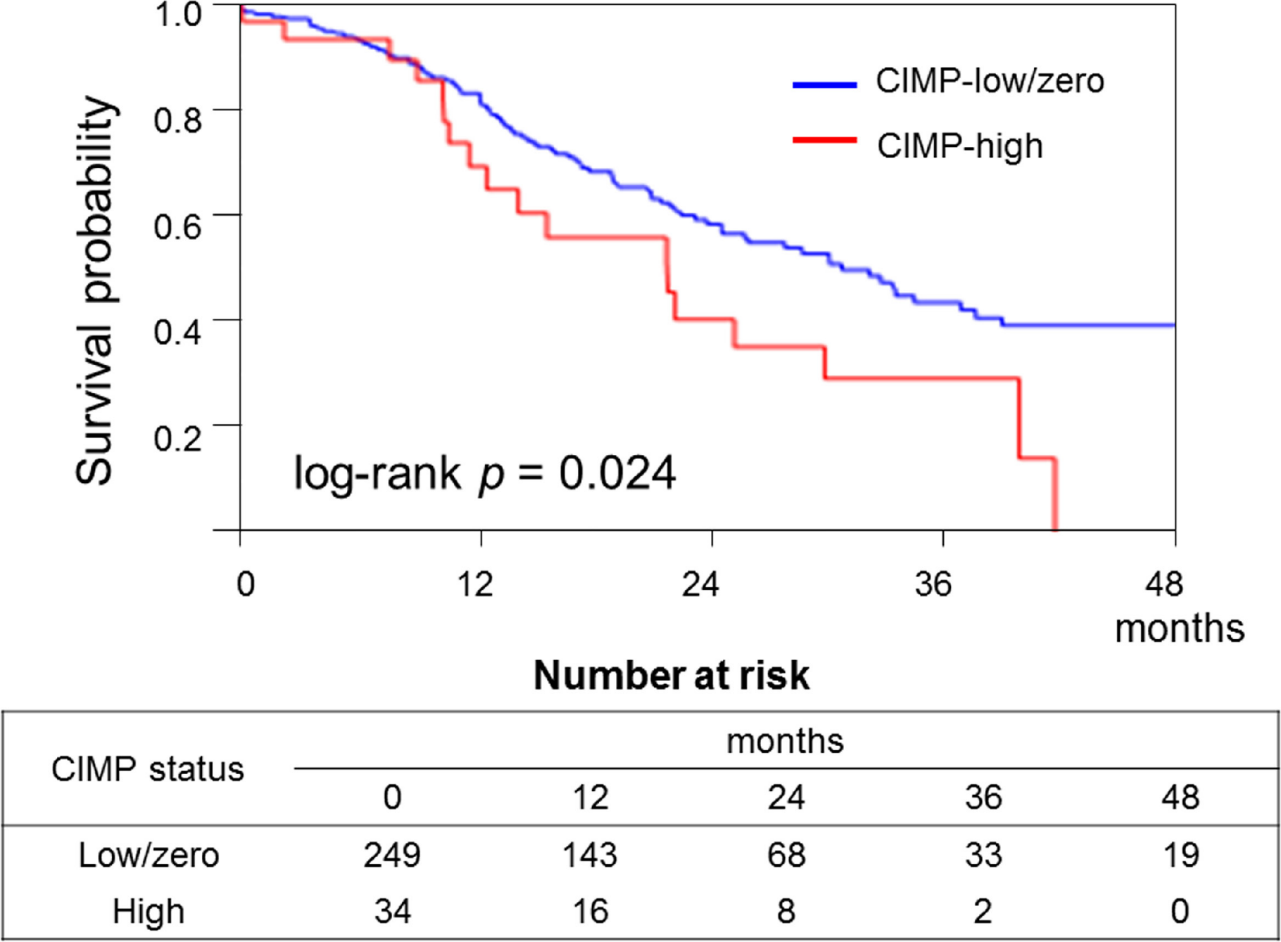
Kaplan–Meier curves of cancer-specific survival of patients with pancreatic cancer according to CpG island methylator phenotype (CIMP) status In the Kaplan–Meier analysis, CIMP-high status significantly correlated with shorter survival (log-rank *p* = 0.024).

In the Kaplan–Meier analysis, high expression levels of miR-21 (log-rank *p* = 0.0025; Figure 3A), miR-31 (log-rank *p* = 0.0003; Figure 3B), and CIMP-high status (log-rank *p* = 0.024; Figure 4) significantly correlated with shorter survival. In contrast, neither *KRAS* mutations nor miR-143 was associated with patient survival (data not shown).

### Multivariate Cox regression analysis in patients with pancreas cancer

In the univariate Cox regression analysis, compared to *Fusobacterium* species-negative cases, significantly higher mortality rates were observed among *Fusobacterium* species-positive cases [hazard ratio (HR): 1.91; 95% confidence interval (CI): 1.04–3.24; *p* = 0.037; Table 2].

**Table 2:** The association between tumor *Fusobacterium* species status and mortality of patients with pancreatic cancer

*Fusobacterium* species status	Total N	*Fusobacterium* species status	
		UnivariateHR (95% CI)	Multivariatestage-stratifiedHR (95% CI)
Negative	258	1 (referent)	1 (referent)
Positive	25	1.91 (1.04–3.24)	2.16 (1.12–3.91)
*P*		0.037	0.023

The multivariate, stage-stratified Cox model included the *Fusobacterium* species variable adjusted by gender; age at diagnosis; tumor size; tumor location; year of diagnosis; *KRAS* mutation status; CIMP status and expression levels of microRNA-21, microRNA-31, and microRNA-143. A backward stepwise elimination with a threshold of *p* = 0.10 was used to select variables in the final model. Of the variables, *Fusobacterium* species, CIMP status, and expression level of microRNA-31 were included in the final model.

CI, confidence interval; CIMP, CpG island methylator phenotype; HR, hazard ratio

A multivariate model initially included gender, age at diagnosis, tumor size, tumor location, year of diagnosis, *KRAS* mutation, CIMP status and miR-21, miR-31, and miR-143 expression levels. Similarly, compared to *Fusobacterium* species-negative cases, an independent association with poorer prognosis was observed in *Fusobacterium* species-positive cases in the multivariate analyses of cancer-specific survival (HR: 2.16; 95% CI: 1.12–3.91; *p* = 0.023; Table 2).

## DISCUSSION

In the present study on patients with pancreatic cancer who underwent surgical treatment, we tested whether *Fusobacterium* species exist in pancreatic cancer tissue. The detection rate of *Fusobacterium* species in pancreatic cancer tissue specimens in the present study was 8.8%. Moreover, we found that *Fusobacterium* species status of pancreatic cancer tissue specimens was independently associated with worse prognosis. Therefore, our data suggest that *Fusobacterium* species exist in pancreatic cancer tissue and may be related to the malignant potential. Although our data need to be confirmed in other (independent) data sets, tumor *Fusobacterium* species status may serve as a prognostic biomarker of pancreatic cancer.

At present, the pathogenesis of pancreatic cancer is poorly understood. With respect to the association of bacterial infections with pancreatic cancer, another study showed that a history of periodontal disease is associated with an increased risk of pancreatic cancer in a prospective cohort study [10]. Among the oral bacteria, *Porphyromonas gingivalis* (*P. gingivalis*), a pathogen responsible for periodontal disease, is associated with pancreatic carcinogenesis [6]. In addition, a high level of antibodies to *P. gingivalis* in serum correlates with a lower risk of pancreatic cancer [7]. According to culture methods, the microbiota isolated from the pancreas has similarities to the oral microbiota, particularly in cases of pancreatitis [6, 22]. Moreover, the spread of oral bacteria to the pancreas via dissemination has been documented for both animal models and human subjects [7, 15, 31].

Aside from periodontal disease, the genus *Fusobacterium* species is associated with inflammatory disorders of the gastrointestinal tract [16–19], with pancreatic abscesses [22, 25], hepatic abscesses [20, 21], cerebral abscesses [23] and Lemierre's syndrome [32]. These data suggest that oral *Fusobacterium* species may reach the pancreas through the circulation (i.e., blood or lymphatic vessels). Furthermore, multiple studies have shown that the oral microbiota overlaps with the gastrointestinal tract microbiota; these data suggest that there are multiple avenues for the dissemination of oral bacteria within the body [7, 33, 34]. Transductal transmission from the biliary tract (either ascending or descending) has also been considered [31, 35]. Further research is needed to identify the route of *Fusobacterium* species' spread to the pancreas.

Recent studies showed that *Fusobacterium* species are detected in gastrointestinal tract cancers, such as gastric cancer [34] and colorectal cancer [26, 29, 30]; however, published studies have not examined the relationship of *Fusobacterium* species with the cancer outcome in a large sample (*n* > 100) of such patients. In the present study, we found that tumor *Fusobacterium* species status is independently associated with shorter survival in patients with pancreatic cancer. To the best of our knowledge, this is the first report to demonstrate an association of tumor presence of *Fusobacterium* species with the outcome of pancreatic cancer. Although no significant association was found, *Fusobacterium* species were highly detected in pancreatic tail cancer (4/18; 22%) than in head (16/203; 7.9%) or body cancer (5/62; 8.0%). The high prevalence of *Fusobacterium* species in pancreatic tail cancers remains uncertain. One possible explanation for this result is the difference of the vascular supply between the pancreatic tail and head or body. Moreover, in the tumor *Fusobacterium* species-positive cases, *Fusobacterium* species were detectable in 28% of the paired specimens of normal tissues, suggesting that these bacteria may play a role in the pathogenesis of pancreatic cancer. Our findings should help to elucidate the details of pancreatic carcinogenesis and could lead to the development of a new diagnostic or therapeutic method (i.e., eradication) for patients with pancreatic cancer.

Our study has some limitations because of its cross-sectional (observational) design and the risk that an unknown bias (e.g., the selection bias) may confound the results. Furthermore, our analysis does not include treatment data; thus, another unknown bias, e.g., differential treatment assignment, may skew the results. In addition, we excluded cases without available tumor tissue: another possible bias. Nevertheless, the results of our regression analyses are adjusted for potential confounders, including disease stage, year of diagnosis, CIMP status, and miR-21 and miR-31 expression levels.

The role of *Fusobacterium* species in pancreatic carcinogenesis remains unknown. Recent studies showed that *Fusobacterium* species increase production of reactive oxygen species (ROS) and inflammatory cytokines (e.g., IL-6 and TNF) in colorectal cancer [30]. Inflammation and ROS can cause epigenetic silencing of the mismatch repair protein MLH1 [36]. Accordingly, we assessed here the possible association of tumor *Fusobacterium* species status with epigenetic alterations, such as *MLH1* methylation and CpG island methylator phenotype (CIMP) in pancreatic cancer.

The term “CIMP” has been repeatedly used over the past decade to describe CpG island promoter methylation in various human malignancies, including pancreatic cancer [12, 28, 37–39]. In contrast to colorectal cancer research, in the field of pancreatic research, CIMP is an unfamiliar term. One study showed that CIMP-high status is observed in five of 36 (14%) pancreatic cancers; however, there is no significant association between the CIMP status and the prognosis [12]. In contrast, our present data show that CIMP-high status (according to methylated promoters that are widely used in colorectal cancer research), and *MLH1* methylation are detected in 12% and 3.9% of pancreatic cancers. Although neither CIMP status nor *MLH1* methylation correlate with tumor *Fusobacterium* species status, our results show that the CIMP-high status is associated with an unfavorable prognosis of pancreatic cancer. CIMP is believed to be useful not only for molecular characterization but also for the assessment of a response to treatment in a variety of human cancers [37]. Therefore, further research is needed to clarify the role of epigenetic alterations in pancreatic cancer.

MicroRNAs constitute a class of small non-coding RNA molecules that function as post-transcriptional gene regulators and have been increasingly recognised as biomarkers of various human cancers [2, 5, 8, 9, 40–42]. Certain microRNAs are induced during the macrophage inflammatory response and have the ability to regulate host-cell responses to pathogens [43]. In addition, pathogens themselves may regulate microRNA expression [44]. MicroRNAs influence networks that control innate and adaptive immunity and apoptosis by regulating signaling pathways [43]. Previous studies suggest that cells can secrete microRNAs that can be delivered into recipient cells where they can change gene expression [45, 46]. Accordingly, it is possible that immune system-related microRNAs can travel to the pancreas, even if the pathogens are located elsewhere. Nevertheless, no previous studies have reported an association between microbiota and microRNA expression in gastrointestinal cancer.

With regard to microRNA expression levels in pancreatic cancer, a recent study demonstrated that miR-21, miR-31 [2, 9, 11], and miR-143 expression [47] levels are significantly associated with higher stage or worse survival. Therefore, we analyzed the association between tumor *Fusobacterium* species status and miR-21, miR-31, or miR-143 expression levels in pancreatic cancer. No significant associations were found. In line with previous studies [2, 9, 11], our present data also show that miR-21 and miR-31 expression levels are related to shorter survival among patients with pancreatic cancer. Thus, these microRNAs could be diagnostic biomarkers or therapeutic targets in patients with pancreatic cancer.

In conclusion, *Fusobacterium* species were detected in pancreatic cancer tissue. Although no significant association was found between *Fusobacterium* species status and molecular alterations of pancreatic cancers, the tumor *Fusobacterium* species status was independently associated with worse prognosis of pancreatic cancer. Thus, our data suggest that tumor *Fusobacterium* species is a promising biomarker of pancreatic cancer and could open up exciting opportunities to improve our understanding of the pathogenesis of this fatal disease.

## MATERIALS AND METHODS

### Patients and cancer tissue specimens

We collected FFPE tissue specimens of 302 pancreatic cancers [pancreatic ductal adenocarcinomas (PDACs)] of patients who underwent surgical treatment at Sapporo Medical University Hospital, Teine Keijinkai Hospital or Otaru Municipal Hospital between 2003 and 2013. To avoid the selection bias as much as possible, we collected the consecutive FFPE cancer tissue specimens. To clarify the association between *Fusobacterium* and survival in patients with pancreatic cancer, we limited the patients who received adjuvant chemotherapy to those treated with gemcitabine or 5-FU (including S-1). Patients who were treated with chemotherapy or radiotherapy prior to resection were excluded.

Histological data on pancreatic cancer tissue specimens were evaluated by pathologists (T.S. and T.H.) who were blinded to the clinical and molecular information. Cancer-specific survival was defined as the period from the surgical treatment of pancreatic cancer to death or last follow up. The patients were followed until death or until July 2014, whichever came first. Informed consent was obtained from all the patients before specimen collection. This study was approved by the institutional review boards of the three participating institutions and complied with the tenets of the Helsinki Declaration. Our analysis of the pancreatic cancer tissue specimens is fully compliant with the REMARK guidelines [48].

### DNA extraction and quantitative PCR for *Fusobacterium* species

Genomic DNA was extracted from FFPE tissue specimens using QIAamp DNA FFPE Tissue Kit (Qiagen, Valencia, CA, USA). We used custom-made TaqMan primer/probe sets (Applied Biosystems, Foster City, CA, USA) specific for *Fusobacterium* species (the sequences are available upon request). The cycle threshold (Ct) values for *Fusobacterium* species were normalized to prostaglandin transporter (PGT) in each reaction as previously described [27, 49]. The PCR mix consisted of 4 μl of genomic DNA (20 ng/μl), 10 μl of 1× TaqMan Environmental Master Mix 2.0 (Applied Biosystems), 1.0 μl of *Fusobacterium* species-specific 20× TaqMan primer and probe sets, 1.0 μl of the PGT primer and probe sets and 4 μl of microbial-DNA-free water (Qiagen). The conditions for quantitative PCR were as follows: 95°C for 10 min, followed by 40 cycles of 95°C for 15 sec and 60°C for 60 sec. The SDS v1.4 software (Applied Biosystems) was used for comparative analysis of the cycle thresholds (ΔCt).

### Pyrosequencing of *KRAS*, *NRAS*, *BRAF*, and *PIK3CA* and analysis of MSI

Genomic DNA was used for PCR and targeted pyrosequencing of *KRAS* (codon 12, 13, 61, or 146), *NRAS* (codon 12, 13, or 61), *BRAF* (*V600E*), and *PIK3CA* (exon 9 or 20) as described previously [38, 50]. MSI analysis was performed using two markers (BAT25 and BAT26) as described previously [50].

### Sodium bisulfite treatment and real-time PCR (MethyLight) to assess promoter methylation of *CACNA1G*, *CDKN2A* (*p16*), *IGF2*, *MLH1*, and *RUNX3*

Bisulfite modification of genomic DNA was performed using the BisulFlash™ DNA Modification Kit (Epigentek, Brooklyn, NY, USA) [39]. DNA methylation was quantified in five promoters [*CACNA1G, CDKN2A* (*p16*), *IGF2*, *MLH1*, and *RUNX3*] using Real-Time PCR (MethyLight). CIMP-high status was defined as the presence of three/four or more methylated promoters [*CACNA1G, CDKN2A* (*p16*), *IGF2*, and *RUNX3*], and CIMP-low/zero was defined as the presence of zero/four to two/four methylated promoters as described previously [39].

### RNA extraction and quantitative reverse transcription-PCR (RT-PCR) of miR-21, miRNA-31, and miRNA-143

Total RNA was extracted from FFPE tissues using the miRNeasy FFPE Kit (Qiagen). MiR-21-5p, miR-31-5p, and miR-143-3p expression levels were analyzed by quantitative RT-PCR using the TaqMan MicroRNA Reverse Transcription Kit (Applied Biosystems) and TaqMan microRNA Assays (Applied Biosystems) as previously described [50]. U6 small nuclear RNA (snRNA; RNU6B; Applied Biosystems) served as an endogenous control. We defined high expression level groups of miR-21 and miR-31 as the fourth level (Q4) in a quartile as described previously [50]. In contrast, low expression level group of miR-143 was defined as the first level (Q1) in a quartile.

### Statistical analysis

The JMP (version 10) and SAS (version 9) software applications were used for all calculations (SAS Institute, Cary, NC, USA). To assess associations among the clinical, pathological and molecular characteristics, we have used either the χ^2^ test for gender; tumor location in pancreas; tumor size; lymph node invasion; year of diagnosis; *KRAS* mutation; expressions of miR-21, miR-31, and miR-143; *MLH1* methylation and CIMP status or Fisher's exact test for disease stage (UICC classification). To compare mean patient ages, *t*-test was used.

The Kaplan–Meier method and log-rank test were performed to assess the association between *Fusobacterium* species status of pancreatic cancer and patient mortality. In the analysis of cancer-specific mortality, deaths from causes other than pancreatic cancer were censored. To adjust the results for confounders, we used multivariate Cox proportional hazards regression models to calculate HR according to tumor *Fusobacterium* species status of pancreatic cancer. We included disease stages (I, II, III, IV, or unknown) as a stratifying variable using the “strata” option in the SAS “proc phreg” command. The multivariate model initially included gender (male versus female), age at diagnosis (continuous variable), tumor size (<2, 2–4, 4–6, or ≥6 cm), tumor location in the pancreas (head, body or tail), year of diagnosis (continuous variable), *KRAS* mutation status (in codon 12/13/61/146; present versus absent), CIMP status (CIMP-high versus CIMP-low/zero), and miR-21, miR-31, and miR-143 expression levels (high versus low expression levels). To avoid overfitting, variables in the final model were selected using backward stepwise elimination with a threshold of *p* = 0.10.

## Abbreviations

CI: confidence interval
FFPE: formalin-fixed paraffin-embedded
CIMP: CpG island methylator phenotype
HR: hazard ratio
IBD: inflammatory bowel disease
miR-21: microRNA-21
miR-31: microRNA-31
miR-143: microRNA-143
MSI: microsatellite instability
PDAC: pancreatic ductal adenocarcinoma
RT-PCR: reverse transcription-PCR
SD: standard deviation
UICC: Unio Internationalis Contra Cancrum
